# DNA Damage Responses following Exposure to Modulated Radiation Fields

**DOI:** 10.1371/journal.pone.0043326

**Published:** 2012-08-17

**Authors:** Colman Trainor, Karl T. Butterworth, Conor K. McGarry, Stephen J. McMahon, Joe M. O’Sullivan, Alan R. Hounsell, Kevin M. Prise

**Affiliations:** 1 Centre for Cancer Research and Cell Biology, Queen’s University Belfast, Belfast, Northern Ireland; 2 Radiotherapy Physics, Northern Ireland Cancer Centre, Belfast Health and Social Care Trust, Belfast, Northern Ireland; 3 Clinical Oncology, Northern Ireland Cancer Centre, Belfast Health and Social Care Trust, Belfast, Northern Ireland; University Health Network, Canada

## Abstract

During the delivery of advanced radiotherapy treatment techniques modulated beams are utilised to increase dose conformity across the target volume. Recent investigations have highlighted differential cellular responses to modulated radiation fields particularly in areas outside the primary treatment field that cannot be accounted for by scattered dose alone. In the present study, we determined the DNA damage response within the normal human fibroblast AG0-1522B and the prostate cancer cell line DU-145 utilising the DNA damage assay. Cells plated in slide flasks were exposed to 1 Gy uniform or modulated radiation fields. Modulated fields were delivered by shielding 25%, 50% or 75% of the flask during irradiation. The average number of 53BP1 or γH2AX foci was measured in 2 mm intervals across the slide area. Following 30 minutes after modulated radiation field exposure an increase in the average number of foci out-of-field was observed when compared to non-irradiated controls. In-field, a non-uniform response was observed with a significant decrease in the average number of foci compared to uniformly irradiated cells. Following 24 hrs after exposure there is evidence for two populations of responding cells to bystander signals in-and out-of-field. There was no significant difference in DNA damage response between 25%, 50% or 75% modulated fields. The response was dependent on cellular secreted intercellular signalling as physical inhibition of intercellular communication abrogated the observed response. Elevated residual DNA damage observed within out-of-field regions decreased following addition of an inducible nitric oxide synthase inhibitor (Aminoguanidine). These data show, for the first time, differential DNA damage responses in-and out-of-field following modulated radiation field delivery. This study provides further evidence for a role of intercellular communication in mediating cellular radiobiological response to modulated radiation fields and may inform the refinement of existing radiobiological models for the optimization of advanced radiotherapy treatment plans.

## Introduction

The delivery of clinical radiotherapy is based upon the assumption that radiobiological response within the target volume is proportional to the dose delivered [1]. During the delivery of advanced radiotherapy techniques such as intensity modulated radiation therapy (IMRT), modulated beams are utilised to increase conformity of dose delivered to the targeted tumour volume. However, there is increasing experimental evidence of differential cellular responses to modulated fields occurring particularly outside the primary treatment field (out-of-field areas).

Several reports have highlighted significant differences in cell survival [2]–[7] and DNA damage response [6] following modulated field exposure. Recent investigations from our own laboratory outlined an important role for intercellular communication on out-of-field cell survival after exposure to a modulated 6 MV photon beam [4]. Decreased survival was observed out-of-field after exposure to a modulated radiation field with evidence of increased levels of survival in-field (i.e. within the primary treatment field). This response could be prevented by physically inhibiting communication between the cell populations. Further evidence from our laboratory demonstrated intercellular communication between the in-and out-of-field cell populations as central mediator of response [5]. A significant decrease in cell survival was observed out-of-field for several cell lines of different radiosensitivity following exposure to modulated radiation fields. Similarly to Butterworth *et al* [4], the response could be abrogated by physically inhibiting cellular secreted intercellular communication between the in-and out-of-field cellular populations. A role for nitric oxide in mediating response was also observed with addition of Aminoguanidine, a nitric oxide synthase (iNOS) inhibitor, or cPTIO, a non-specific NO scavenger, causing increased survival within the out-of-field region. By determining the mean nuclear γ-H2AX-associated fluorescence intensity Syme *et al* [6] provided evidence for an enhancement of DNA damage within the penumbra and blocked regions in normal fibroblast cells compared to an open-beam irradiation, highlighting a role for differences in beam quality within areas out-of-field.

Recent investigations indicate a role for the radiation induced bystander effect in mediating cellular responses to modulated radiations fields [2]–[7]. The radiation induced bystander effect, defined as a radiobiological response in cells not directly traversed by a radiation field, is driven through either secretion of factors into the surrounding environment or direct cell-to-cell contact [8]. The effect has been observed at several end-points including cell survival [9]–[11], micronuclei formation [12]–[13] and DNA damage induction [14]–[20].

Double strand breaks (dsb) are generally accepted to be the most significant form of DNA damage lesion as they lead to both cell death and stable genetic alterations if left unrepaired. Utilising antibodies directed against p53 binding protein 1 (53BP1) and phosphorylated histone H2AX (γH2AX) allows for accurate measurements of DNA damage lesions. 53BP1 is a member of the BRCT (BRCAI C-Terminal) repeat family and has an important role in the phosphorylation of multiple Ataxia telangiectasia mutated (ATM) substrates during the DNA damage response. It has been shown that both 53BP1 and γH2AX form foci rapidly after irradiation exposure at sites of DNA damage with the number of foci strongly correlating with the number of DNA dsb [21]–[22].

Induction of DNA damage foci in bystander cells has been reported previously [14]–[20]. Using a 1 cGy dose of alpha-particles to irradiate 50% of a mylar-based dish Han *et al* [16] observed induction of γH2AX within AG0-1522 cells present in both irradiated and non-irradiated bystander areas. The induction of DNA damage was seen to reach a maximum 30 minutes following irradiation. *In situ* visualization of dsb by Hu *et al* [17] showed a rapid increase in DNA dsb 2 minutes after irradiation within exposed and non-irradiated bystander areas. 30 minutes following exposure, a 2-fold increase in dsb was observed when compared with sham irradiated controls. Tartier *et al* [19] observed induction of 53BP1 foci within bystander Hela cells utilising a microbeam approach. By carrying out cytoplasmic irradiations they observed that induction of foci within bystander cells is not dependent upon nuclear irradiation. Addition of irradiated conditioned media to non-irradiated bystander cells has also shown to induce 53BP1 foci within glioma and fibroblast cells [20]. Similar to γH2AX bystander foci [15], it was observed that the induction of 53BP1 bystander foci was dependent on Ataxia telangiectasia and Rad3-related protein (ATR) function and not ATM or DNA-dependent protein kinase (DNA-PK).

The purpose of the present study was to determine the spatial distribution of DNA damage both in-and out-of-field following exposure to a modulated radiation field delivered by shielding 25%, 50% or 75% of the cell population. Fluorescent detection of 53BP1 and γH2AX foci was utilised as a marker of DNA damage following irradiation. The time kinetics of the DNA damage responses were investigated along with the role of intercellular communication at several spatial positions. The frequency distribution of 53BP1 foci was determined in the presence and absence of Aminoguanidine to determine the role of reactive nitrogen species on residual DNA damage in regions out-of-field.

## Materials and Methods

### Cell Culture

AG0-1522B cells [5] were obtained from Coriell Institute for Medical Research (Camden, NJ, USA) and grown in Eagle’s Minimum Essential Medium with deoxyribonucleosides and deoxyribonucleotides (Lonza, UK) supplemented with 20% fetal bovine serum and 100 µg/ml streptomycin (Gibco, UK). DU-145 [5] cells were obtained from ATCC LGC Standards (Middlesex, UK) and grown in RPMI-1640 with L-glutamine (Lonza, UK) supplemented with 10% fetal bovine serum and 100 µg/ml streptomycin (Gibco, UK). All cell lines were maintained at 37°C in a humidified atmosphere of 95% air/5% CO_2_.

### Drug Treatment

To examine the role of cellular secreted factors in the DNA damage response after modulated radiation field exposure, an agent known to inhibit inducible nitric oxide synthase (iNOS) was used. Experiments were conducted under standard culture conditions or in the presence Aminoguanidine (AG) at a concentration of 20 µM. AG was diluted in phosphate-buffered saline to the desired final concentration and added to culture medium 2 hours prior to irradiation and remained in contact with the cells throughout the experiment.

### DNA Damage Analysis by Immunofluorescence Microscopy

Cells were plated at a cell density of 1.5×10^5^ cells per flask and allowed to adhere overnight before irradiation at room temperature (25±2°C). Following irradiation, the cells were incubated at 37°C in 5% CO_2_ in air and 95% humidity and removed for fixation in methanol/acetone (1∶1) at specific time points. DNA damage was detected using the immunofluorescence assay. Cells were permeabilised in 0.5% solution of Triton X-100 in PBS (Sigma, UK) and then blocked with a solution of 0.1% Triton X-100, 5% fetal bovine serum and 2 mg/ml skim milk in PBS. After blocking, cells were incubated with anti-53BP1 rabbit antibody (Novus Biologicals, UK, 1 in 5000) or an anti-phospho-histone H2AX mouse antibody (Millipore, UK, 1in 3000), for one hour at room temperature. Following primary antibody incubation, cells were washed with a 0.1% Triton X-100 in PBS washing buffer and incubated with either Alexa Fluor 488-labelled anti-rabbit IgG secondary antibody (Molecular Probes, UK, 1 in 1000) or Alexa Fluor 488-labelled anti-mouse IgG secondary antibody (Molecular Probes, UK, 1 in 1000) for one hour at room temperature. Cells were washed in PBS and counterstained with 4,6-diamidino-2-phenyindole (DAPI) containing mounting medium for fluorescent microscopy (Vectorshield, UK). Slides were mounted with a 22 mm × 50 mm coverslip and viewed using Zeiss Axiovert 200 M microscope (Carl Zeiss MicroImaging, LLC, North America).

### Experimental Design and Validation of Irradiation Set-up

**Figure 1:**
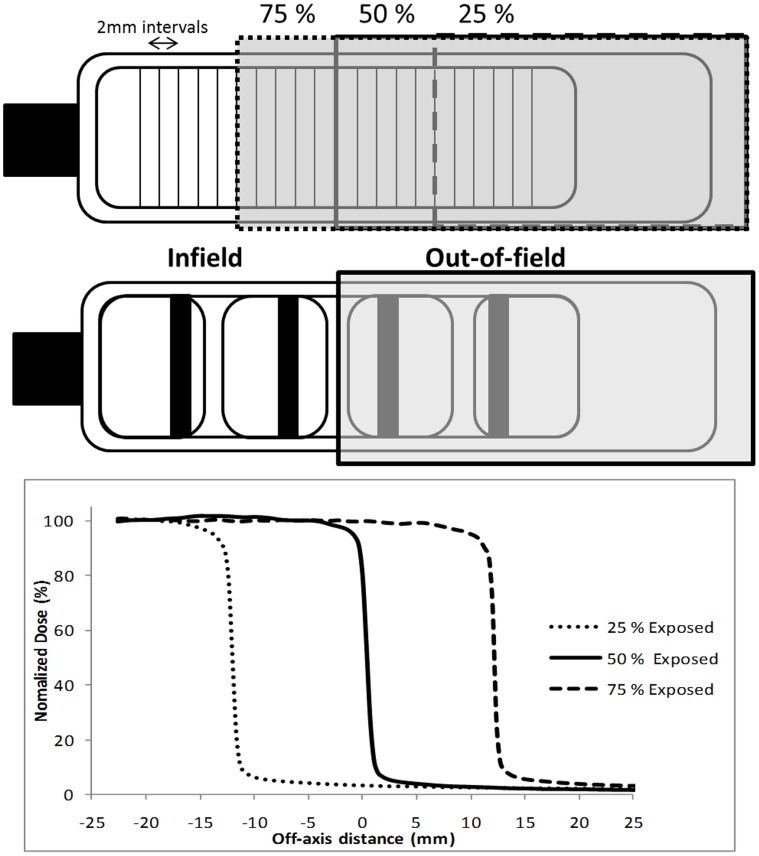
Schematic representation of irradiation set-up and the dose profile for modulated radiation delivery. Cells were irradiated within a slide flask with 25%, 50% or 75% of the slide area under shielding and foci scored in 2 mm intervals from the centre of the flask. Additional investigations into the role of intercellular communication utilised 4 well multichambered slides with cells scored in the specific regions highlighted. Dose profiles were measured using Gafchromic EBT film in the X-Rad 225 kVp for 25% (dotted line) 50% (solid line) and 75% (dashed line) exposures.

DNA damage responses following 1 Gy delivered as a uniform or modulated exposure were determined in a slide flask (Nunc, UK) irradiated using a 2 mm Cu filtered 225 kVp X-ray source (X-Rad 225, Precision, X-ray Inc, USA). Modulated exposures were delivered by shielding a percentage of the flask area with a 13.6×10.4×2.1 cm^3^ block manufactured from a low melting point alloy (MCP96-Mining & Chemical Products Ltd, 1–4 Nielson Road, Finedon Road Industrial Estate, Wellingborough, Northants). During irradiation, cells (covered with a 3 mm layer of culture media (3 mls)) were aligned with the central axis of the beam with the shielding located 2.8 cm above the out-of-field region. For the 50% modulated field the out-of-field area was determined as the 2.6 cm of the flask located under the shielding shown in figure 1. Investigations into the effect of in-field area on foci distribution involved the delivery of a 25% and 75% modulated field. The out-of-field area for 25% and 75% modulated fields was determined as 3.9 cm and 1.3 cm respectively. For each of the investigations unexposed controls were prepared and treated as sham exposures. When analysing DNA damage foci the slides were divided into 2 mm intervals from the centre of the slide and 50 cells were scored for foci within each interval. The average foci number was plotted against distance to generate spatial distribution plots.

For experiments investigating physical inhibition of intercellular communication, millicell EZ slides (Millipore, UK) were utilised as shown in figure 1. In each chamber 3.75×10^4^ cells were plated and allowed to adhere overnight. Slides were exposed to 1 Gy as a modulated field delivered using the same irradiation set-up discussed above. For each of the investigations unexposed controls were prepared and treated as sham exposures. When counting 53BP1 foci distinct areas (4–6 mm and 16–18 mm from the central axis of the flask) in-and out-of-field where chosen to assess DNA damage. Average foci number was plotted for each individual area and compared with DNA damage response when communication was intact.

**Figure 2:**
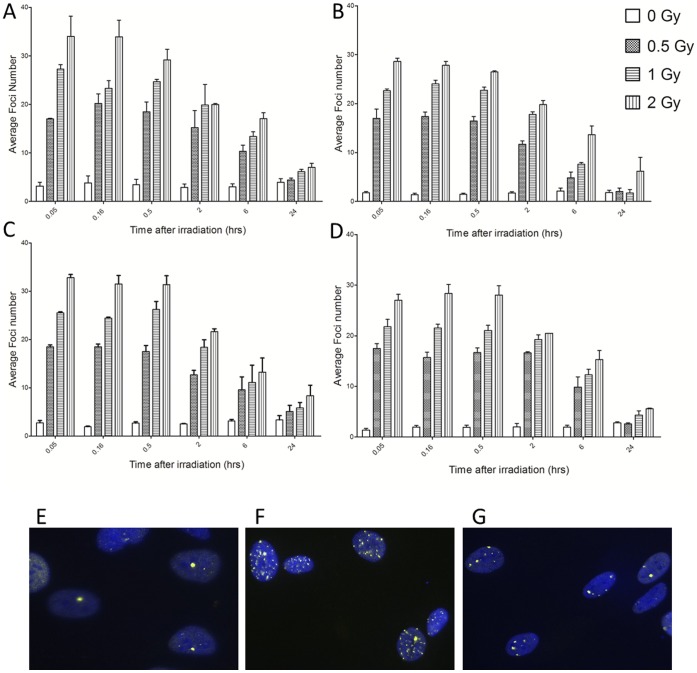
DNA damage induction and repair following exposure to a uniform radiation field. Induction of 53BP1 (A–B) and γ-H2AX (C–D) foci shown for AG0-1522B (A,C) and DU-145 (B,D) cells at 0.05, 0.16, 0.5, 2, 6 and 24 hours following 0.5, 1 and 2 Gy uniform irradiation. Error bars indicate ± standard error of the mean. (E–G) Photographs illustrating induction of 53BP1 foci in non-irradiated (E) AG0-1522B cells compared with uniformly irradiated cells at 30 minutes (F) and 24 hours (G) following a 1 Gy exposure.

Ionisation chamber measurements were taken in conjunction with dosimetric film measurements to determine the scattered dose under the MCP shielding. Gafchromic EBT film (ISP Corp) was cut into the shape of a slide and placed on the underside of a flask. The film was exposed either 2 or 8 Gy and measurements were used to generate a dose profile across three modulated radiation field set-ups (25%, 50%, and 75%) for the 225 kVp X-ray unit (fig. 1). The dose delivered to the out-of-field region of the flask was taken as the average scattered dose to that region. For a 50% modulated field the scattered dose received out-of-field was determined to be 3% of the total dose delivered to the in-field region of the slide flask.

### Statistical Analysis

Statistical errors on values were calculated as the standard error. All experiments were performed at least three times with the data presented as ± standard error in each case. Statistical analysis comparing the averaged foci number was performed with Graphpad Prism V5.01 using the unpaired t-test with significant differences assumed at the level of p<0.05.

In addition to measuring the mean number of foci for in-and out-of-field populations, the distribution of foci was analysed 24 hours following a 50% modulated field exposure. If foci were accumulated randomly within cells, then it would be expected that they would follow a Poisson distribution. This property was tested for foci distributions in two ways, 1) by calculating the dispersion index of the distribution and 2) by statistically testing against a fitted Poisson distribution. Distribution data for in-field area, time kinetics and intercellular communication experiments were not statistically robust for this test and instead are presented as the average foci number which correlates with distribution.

Dispersion index is a measure of the spread of a distribution, defined as σ^2^/μ, where σ is the standard deviation of the population and μ is its mean value. A dispersion index of 1 indicates the population’s spread is equal to that of the Poisson distribution whilst a value less than 1 indicates a more tightly clustered distribution and a value above one indicates a higher degree of spread. Dispersion indices of greater than 1 are often indicative of a population comprised of multiple sub-populations with different means.

Foci distributions were tested for agreement with a Poisson distribution by fitting a Poisson distribution to the observed values using χ^2^ minimisation, and calculating a p-value for agreement using the χ^2^ test.

**Figure 3:**
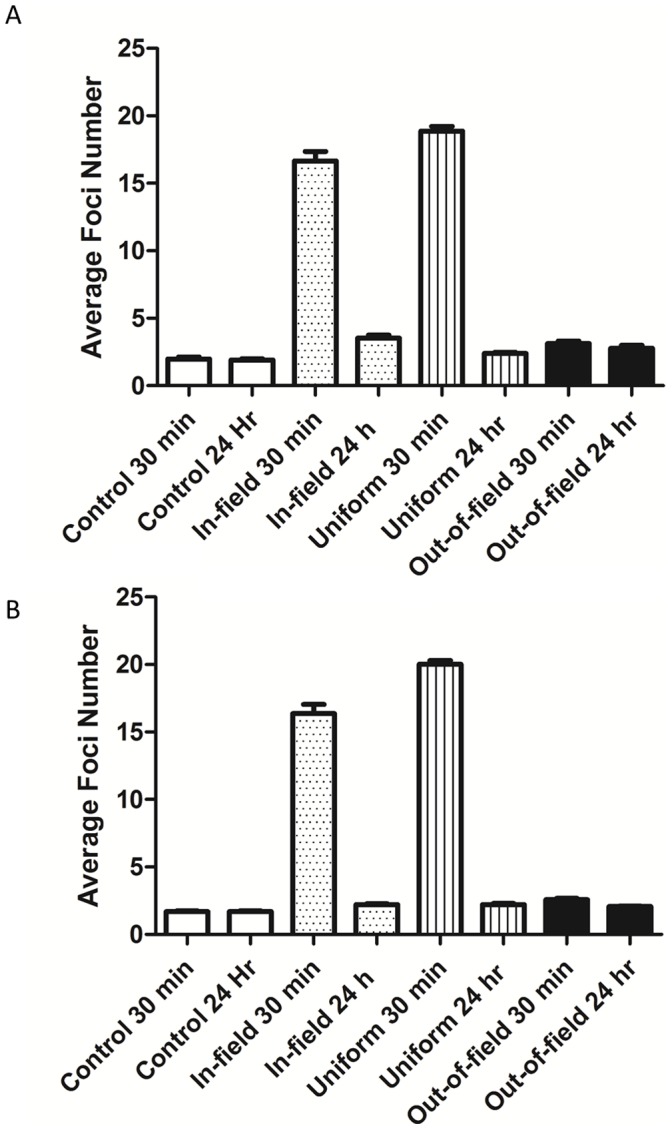
DNA damage induction and repair following exposure to a modulated radiation field. Average 53BP1 foci levels in a uniform (Striped) field compared with the in-field (dotted), out-of-field (solid) and non-irradiated control (open). Data are shown for AG0-1522B cells (A) and DU-145 cells (B) at both 0.5 and 24 hrs after 1 Gy irradiation. When determining average foci number to the in-and out-of-field region a 1 cm central penumbra region was excluded from analysis. Error bars indicate ± standard error of the mean.

## Results

### Induction and Repair of DNA Damage by Measurement of 53BP1 and γH2AX Foci

To determine appropriate experimental conditions for investigating modulated exposures, a series of uniform irradiations at a variety of time points were investigated. AG0-1522B and DU-145 cells were plated on coverslips and exposed to doses of 0.5, 1 and 2 Gy and fixed at several time points (fig. 2). Following uniform irradiation, a rapid induction of 53BP1 (2A–B) and γH2AX (2C–D) foci is observed with the maximum average number of foci observed 30 minutes after exposure at all doses. The response observed was dose dependent as increased dose resulted in increased average foci per cell. A gradual decrease in average number of foci was observed at 2 and 6 hours. After 24 hours, foci levels decreased to levels similar to the non-irradiated control with the exception of the 24 hour samples receiving 2 Gy.

**Figure 4:**
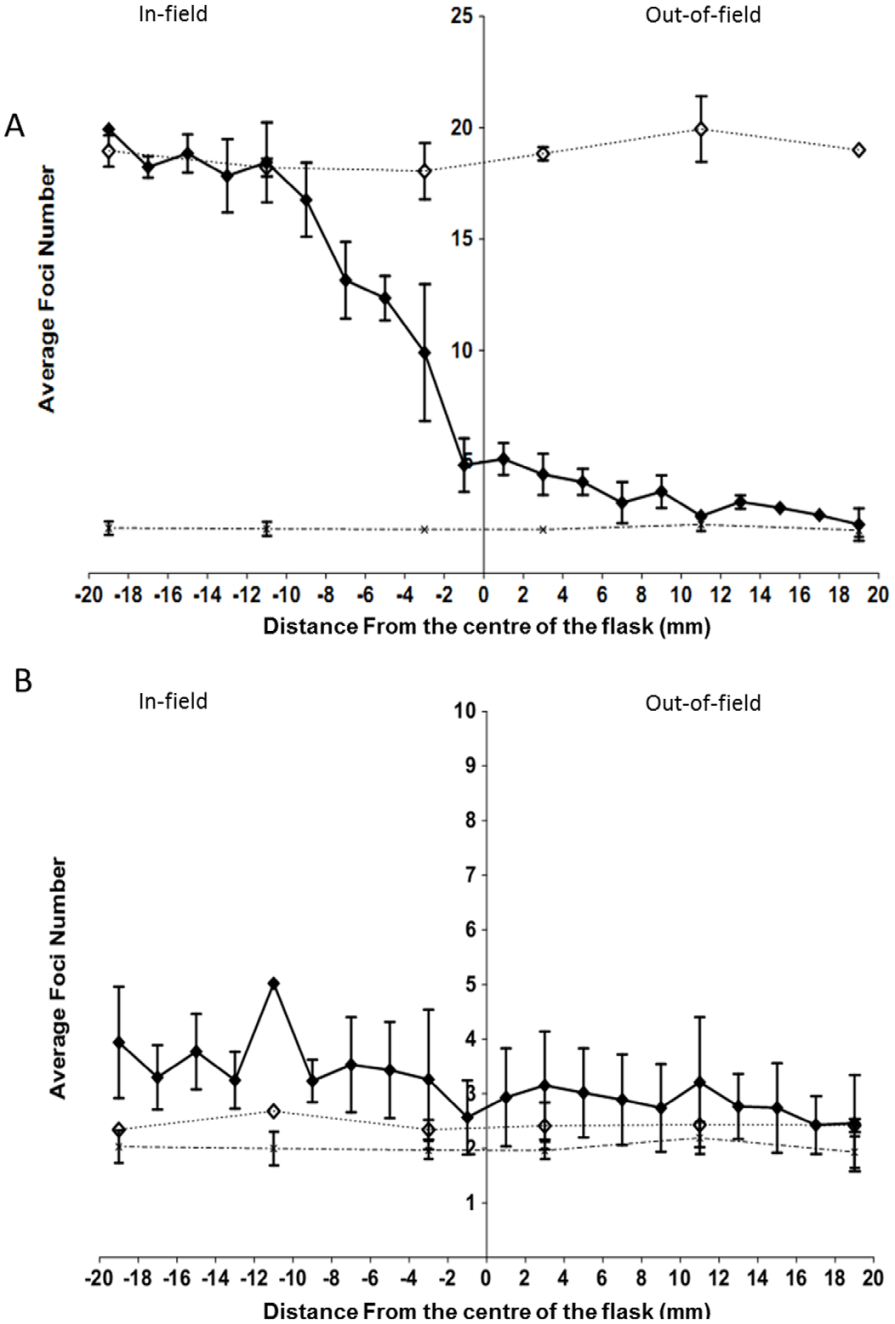
Spatial distribution of 53BP1 foci compared at 2 mm intervals across a slide flask following exposure to uniform or modulated radiation fields. Data are shown for AG0-1522B cells following exposure to a uniform (dotted line) and a modulated radiation field (solid line). The average number of 53BP1 foci were measured 0.5 (A) and 24 hours (B) following either a uniform (⋄) or modulated (⧫) 1 Gy exposure. Non irradiated controls (X) are also included. Error bars indicate ± standard error of the mean.

### Induction and Repair of DNA Damage Following Modulated Radiation Exposure

DNA damage responses following exposure to either uniform or modulated radiation fields were determined in normal human fibroblast cells, AG0-1522B and human prostate cancer cells; DU-145. Comparisons of the average number of 53BP1 foci observed for the primary treatment field (in-field), the shielded region of the flask (out-of-field), the uniform field and non-irradiated control at 30 minutes and 24 hours after irradiation are shown in figure 3. Exposure to a uniform or modulated radiation field resulted in increased 53BP1 foci 30 minutes after irradiation in both cell lines. The average number of foci decreased in the subsequent 24 hours following irradiation. A non-uniform response was observed in-field with a significant decrease (p<0.05) of 11.7% in the average number of foci when compared with the uniform field response 30 minutes after exposure in AG0-1522B cells (fig. 3A). In the subsequent 24 hours following irradiation a significant increase (p<0.05) of 46.3% in the average foci number was observed in-field when compared with uniformly irradiated cells (fig. 3A). At both 30 minutes and 24 hrs the out-of-field region displayed an increase in the average number of foci when compared with non-irradiated controls in AG0-1522B cells.

**Figure 5:**
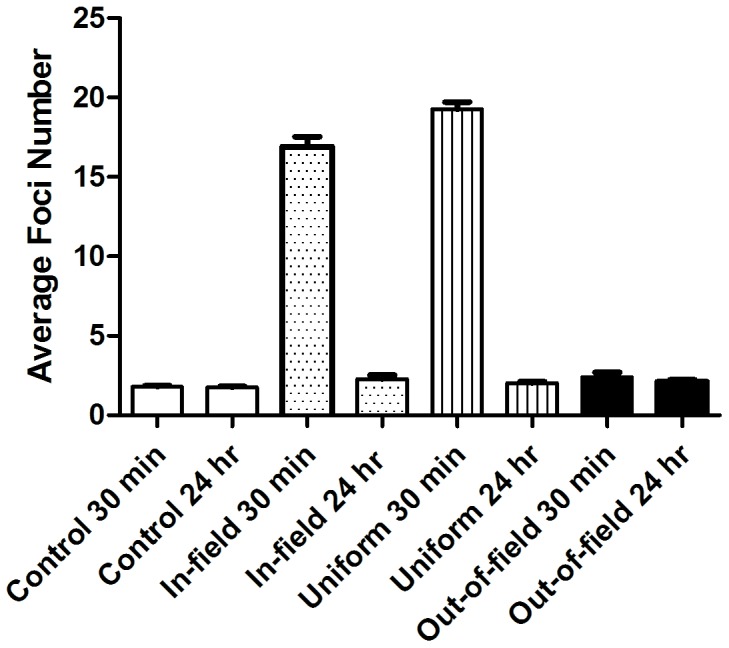
DNA damage induction and repair following exposure to a modulated radiation field measured by γ-H2AX foci. Average γ-H2AX foci levels within a uniform (Striped) field compared with the in-field (dotted), out-of-field (solid) and non-irradiated control (open). Data are shown for AG0-1522B cells at both 0.5 and 24 hrs after 1 Gy irradiation. When determining average foci number to the in-and out-of-field region a 1 cm central penumbra region was excluded from analysis. Error bars indicate ± standard error of the mean.

In DU-145 cells (fig. 3B) a non-uniform response was observed in-field with a significant decrease (p<0.05) of 18.3% in the average number of foci when compared with uniformly irradiated cells 30 minutes after exposure. In the subsequent 24 hours following irradiation no significant difference was observed between cells in-field when compared with the uniform field (fig. 3B). At both 30 minutes and 24 hours following exposure the out-of-field region displayed an increase in the average number of foci when compared with non-irradiated controls.

**Figure 6:**
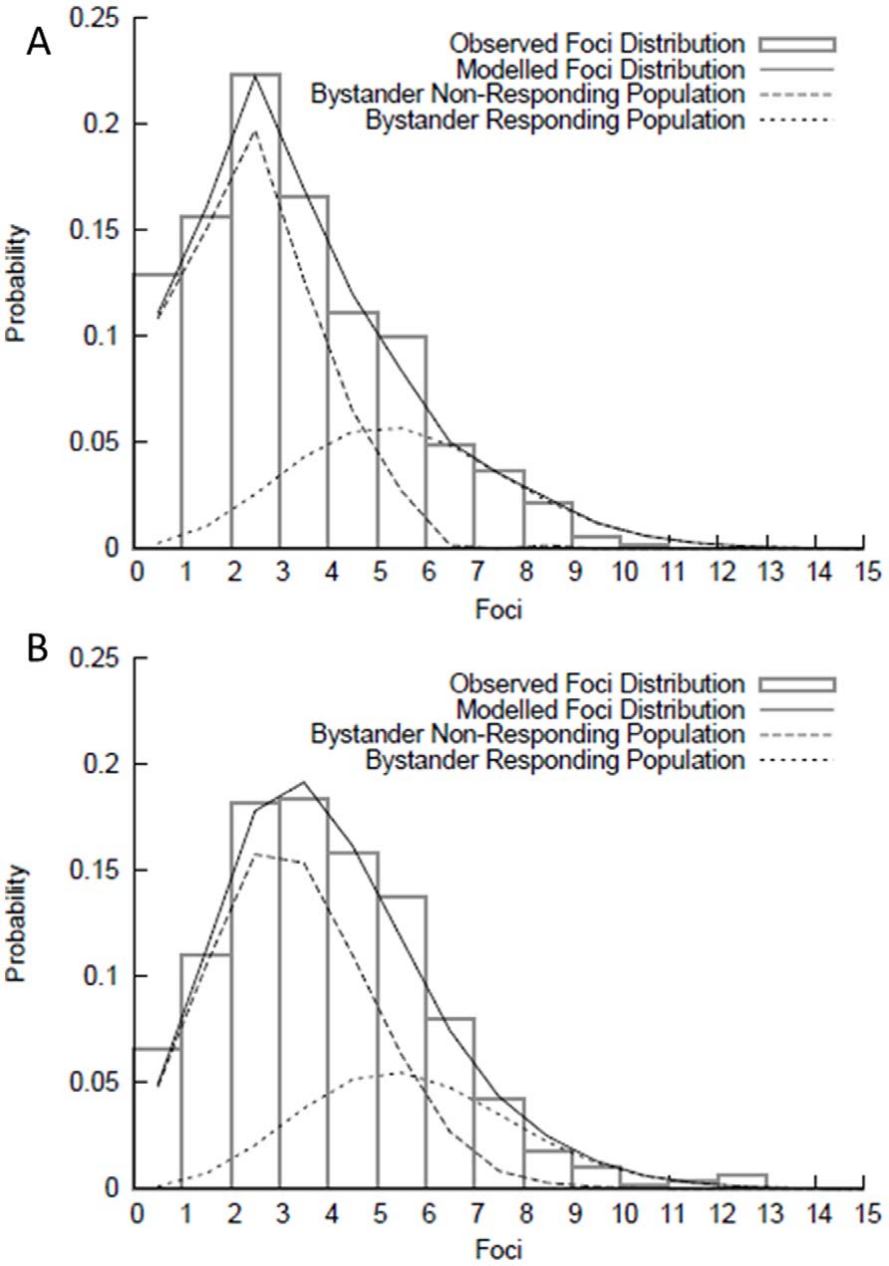
Frequency distribution of 53BP1 foci 24 hours following modulated radiation field. Distributions are shown out-of-field (A) and in-field (B) for AG0-1522B cells (bars). In both cases, these distributions were found to disagree significantly with single-value Poisson distributions. Good agreement was obtained with a model which considered two sub-populations (solid line), made up of one population of non-bystander responding cells (dashed line) and one population of bystander-responding cells (dotted line) which see added foci due to the bystander effect.

### Spatial Distribution of 53BP1 Following Exposure to a Modulated Radiation Field

Spatial distribution profiles of 53BP1 foci for AG0-1522B cells following exposure to a uniform or modulated radiation field are shown in figure 4. A significant decrease (p<0.05) in the average number of foci was observed up to 8 mm from the centre of slide when compared with uniformly irradiated cells 30 minutes following exposure (fig. 4A). Between 10–18 mm in-field the number of 53BP1 foci gradually increased however the average number remained lower than that observed for the uniformly irradiated cells. Elevated levels of 53BP1 were observed out-of-field up to 10 mm from the centre of the slide 30 minutes (fig. 4A) after modulated exposure. Within the subsequent 24 hours (fig. 4B) following irradiation the in-and out-of-field regions displayed an increase in the average number of foci above the non-irradiated control across the entire slide area. The average number of 53BP1 foci was also observed to be significantly increased between 6–18 mm in-field when compared with uniformly irradiated cells.

### Induction and Repair of DNA Damage Following Modulated Radiation Exposure Measured by γH2AX

In order to determine if the response to modulated radiation fields was specific to 53BP1 we investigated the DNA damage marker γH2AX within the AG0-1522B cell line. Comparisons of the average number of γH2AX foci observed for the primary treatment field (in-field), the shielded region of the flask (out-of-field), the uniform field and non-irradiated control at 30 minutes and 24 hours after irradiation are shown in figure 5. As expected, an increase of γH2AX foci was observed 30 minutes following irradiation with a subsequent decrease of the average number of foci 24 hours after exposure. Similarly to 53BP1 foci induction, a non-uniform response was observed in-field with a significant decrease (p<0.05) of 12.2% in the average number of foci when compared with uniformly irradiated cells 30 minutes after exposure. At 24 hours following irradiation an increase of 12.5% in the average foci number was observed in-field when compared with the uniform field exposure. At both time points investigated the out-of-field region displayed an increase in the average number of foci above the non-irradiated control (fig. 5).

**Figure 7:**
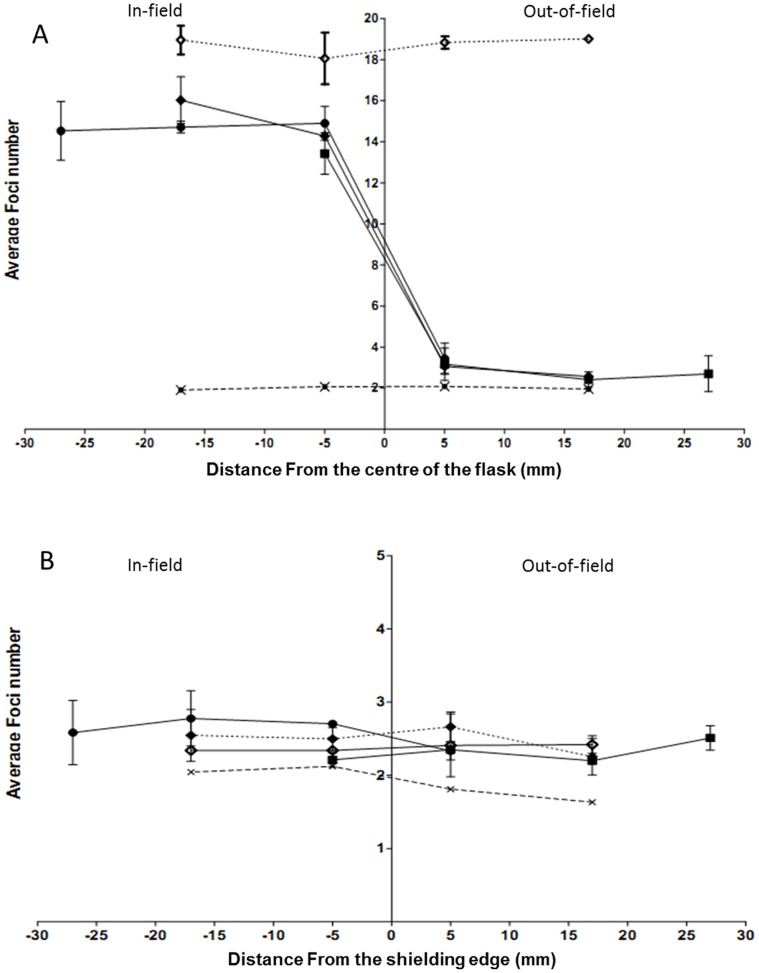
DNA damage induction following variation of in-field area. Spatial distribution of 53BP1 foci 0.5 (A) and 24 hrs (B) within AG0-1522B cell line following modulated exposures in which either 75% (▪) 50% (⧫) or 25% (•) of the flask area was shielded. Levels of 53BP1 following uniform (⋄) irradiation 0.5 and 24 hours and non-irradiated controls (X) are also included. Error bars indicate ± standard error of the mean.

### Comparisons of Distribution of Foci

The distribution of foci in non-irradiated AG0-1522B cells was found to be in good agreement with a Poisson distribution (dispersion of 0.96±0.07, p>0.15). In analysing individual sections from the samples, increased dispersion was observed in many sections of both the in-and out-of-field areas, but these effects were of limited statistical significance. By aggregating all of the 2 mm intervals analysed in-and out-of-field statistically significant observations could be made. By grouping observations together into combined in-and out-of-field populations at 24 hours, it was observed that both distributions had high dispersions, indicating significant deviation from the Poisson distribution observed in the control samples (dispersions of 1.35±0.08 for out-of-field cells, and 1.53±0.10 for in-field cells). Testing these distributions for agreement with a single Poisson distribution also yielded statistically significant disagreements, with p<0.005 for both populations. This suggests that, rather than the bystander effect causing a small, uniform increase in damage in all cells, there is one population of cells which see a significant increase in damage, and another which sees little or no additional damage. This is consistent with investigations by Burdak-Rothkamm *et al* [15] that also observed a sub fraction of bystander cells that have elevated DNA damage foci.

**Figure 8:**
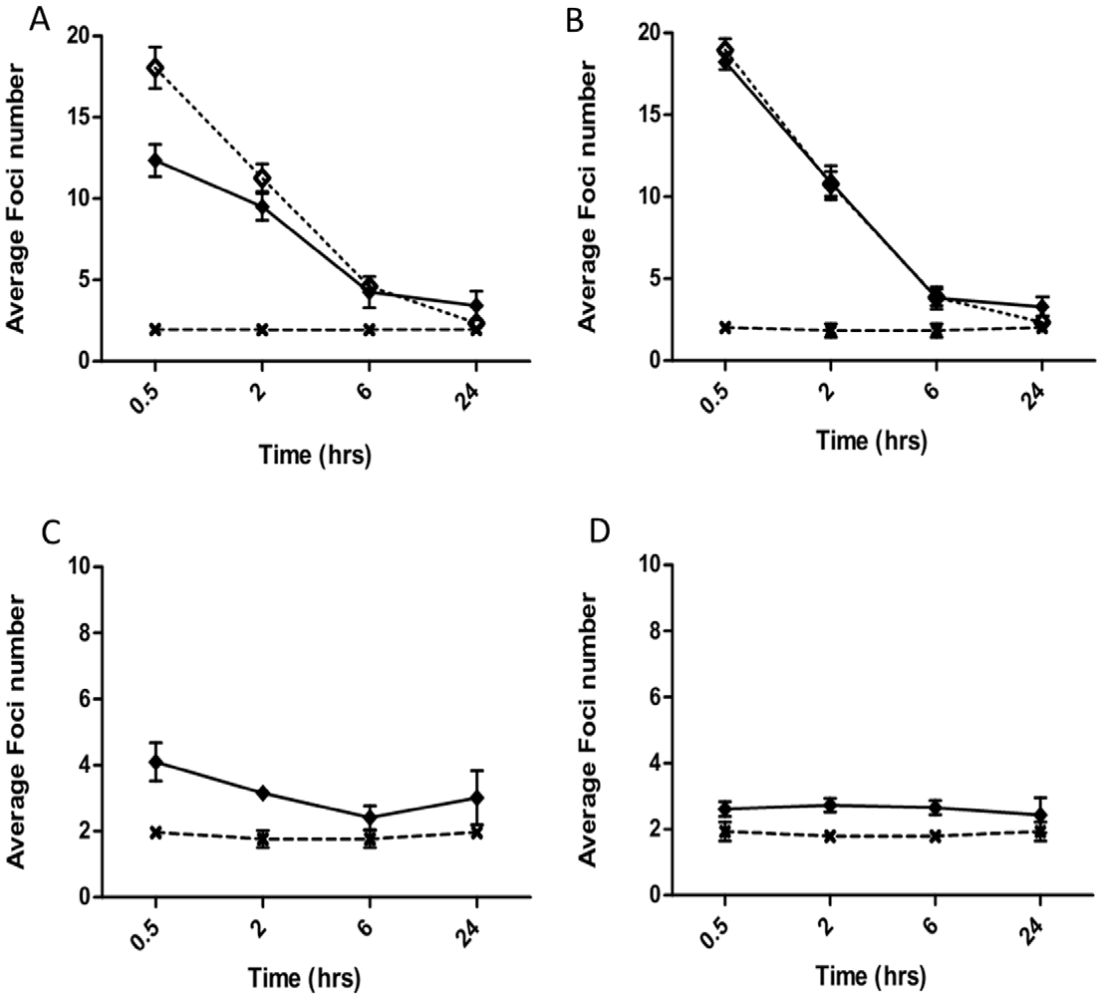
Time kinetics of DNA damage and repair following 1 Gy modulated exposure within AG0-1522B. 53BP1 levels were measured following uniform (⋄) or modulated (⧫) radiation field exposure 4–6 mm (A) and 16–18 mm (B) in-field and 4–6 mm (C) and 16–18 mm (D) in the out-of-field region. Non irradiated controls (X) are also included. Error bars indicate ± standard error of the mean.

This observation was tested by attempting to fit a two-population model to the observed data. Out-of-field, these populations were taken to be non-responding cells, which saw no additional damage, and therefore followed the control distribution, and responding cells, which saw additional, Poisson-distributed damage due to the bystander effect. In-field, the two populations were once again taken to be bystander responders and non-responders, but both populations also saw some additional damage, resulting from the direct radiation exposure. These models were fitted by adjusting three parameters – the fraction of cells which responded to the bystander effect, the amount of foci induced in a bystander-responding cell, and the amount of damage induced by 1 Gy of radiation.

Fitting these values to the modulated field, 1 Gy exposures carried out in this work gave values of 0.32±0.02 for the fraction of cells which respond to the bystander effect, which leads to the induction of 3.1±0.2 additional foci above the non-irradiated control levels with direct radiation damage also leading to additional 0.82±0.08 foci. The predicted curves from this model are plotted in figure 6, together with the observed foci distributions, showing good agreement, suggesting that this two-population model accurately reflects the mechanism of the bystander effect in this system.

**Figure 9:**
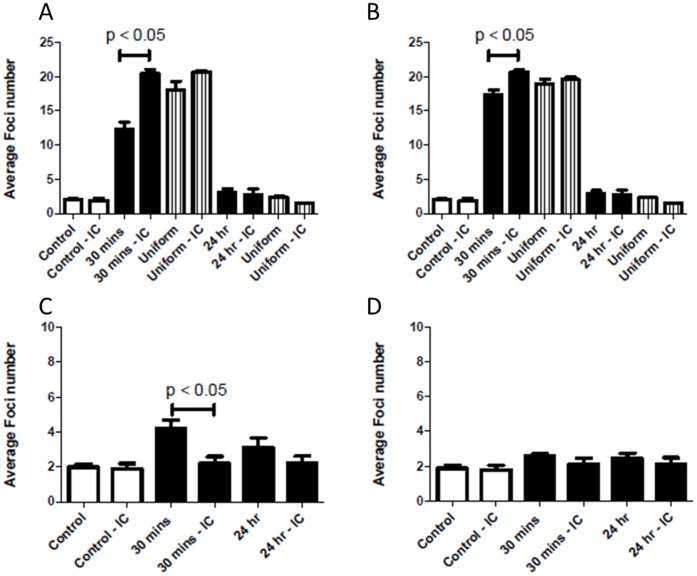
Comparison of DNA damage response following exposure to 1 Gy modulated radiation field with intact or inhibited (−IC) intercellular communication. The average number for 53BP1 foci were measured following uniform (Lined) and modulated (Solid) radiation field exposure at 4–6 mm (A) and 16–18 mm (B) in-field and 4–6 mm (C) and 16–18 mm (D) out-of-field. Non irradiated controls (open) are also included. Error bars indicate ± standard error of the mean.

### The Effect of In-field Area on Foci Distribution

Comparisons of spatial distribution of 53BP1 foci for AG0-1522B cells following modulated exposures in which 25%, 50% or 75% of the slide area was exposed to irradiation are shown in figure 7. Within all modulated radiation fields investigated a non-uniform response was observed in-field 30 minutes following exposure (fig. 7A). The out-of-field region for both the 25 and 75% modulated radiation field displayed elevated levels of 53BP1 above the control cells up to 8 mm from the position of the shielding at 30 minutes. Following 24 hours (fig. 7B) no significant difference was observed in the average number of 53BP1 foci when 25%, 50% or 75% of the flask was irradiated. Each of modulated fields investigated showed an increase in the average number of 53BP1 foci out-of-field when compared with non-irradiated cells at 24 hours.

### Differences in Kinetics of DNA Damage Foci in Relation to Spatial Location

The average numbers of 53BP1 foci measured at specific time points in-and out-of-field compared with uniform and non-irradiated controls are shown in figure 8. In-field the maximum number of 53BP1 foci were measured 30 minutes after irradiation at both 4–6 mm (fig. 8A) and 16–18 mm (fig. 8B) from the centre of the slide. A decrease in the average number of foci was observed within 4–6 mm (fig. 8A) in-field when compared with a uniform exposure at both 30 minutes and 2 hours following irradiation. At 16–18 mm in-field no significant difference was observed when compared with uniformly irradiated cells at 30 minutes and 2 hours. Following 6 hours both regions in-field displayed no significant difference from uniform exposures. An increase in the number of 53BP1 foci was observed at both 4–6 and 16–18 mm in-field when compared with uniformly irradiated cells at 24 hours. Out-of-field the maximum number of 53BP1 foci was observed at 30 minutes for the 4–6 mm region (fig. 8C) and at 2 hours for the 16–18 mm region (fig. 8D). The levels of 53BP1 out-of-field within 4–6 mm remain elevated when compared to non-irradiated controls throughout the subsequent 24 hours following exposure. Within the 16–18 mm out-of-field there is suggestion of elevated average number of foci compared to non-irradiated control however this does not appear to be significant.

### The Role of Intercellular Communication within the Response to Modulated Radiation Fields

The DNA damage responses in-and out-of-field in circumstances where intercellular communication through cellular secreted factors was inhibited or intact are shown in figure 9. For AG0-1522B cells, physical inhibition of cellular secreted factors between the in-and out-of-field cellular populations was shown to abrogate the DNA damage response observed when communication was intact. In-field (fig. 9A–B) a significant increase (p<0.05) in the average of number of foci was observed in conditions where intercellular communication was inhibited. In regions out-of-field (fig. 9C–D) physical inhibition of communication was shown to decrease the average number of foci to levels similar to that of the non-irradiated cells. A significant decrease (p<0.05) in the average number of foci was observed 4–6 mm out-of-field at 30 minutes when intercellular communication was inhibited.

**Figure 10:**
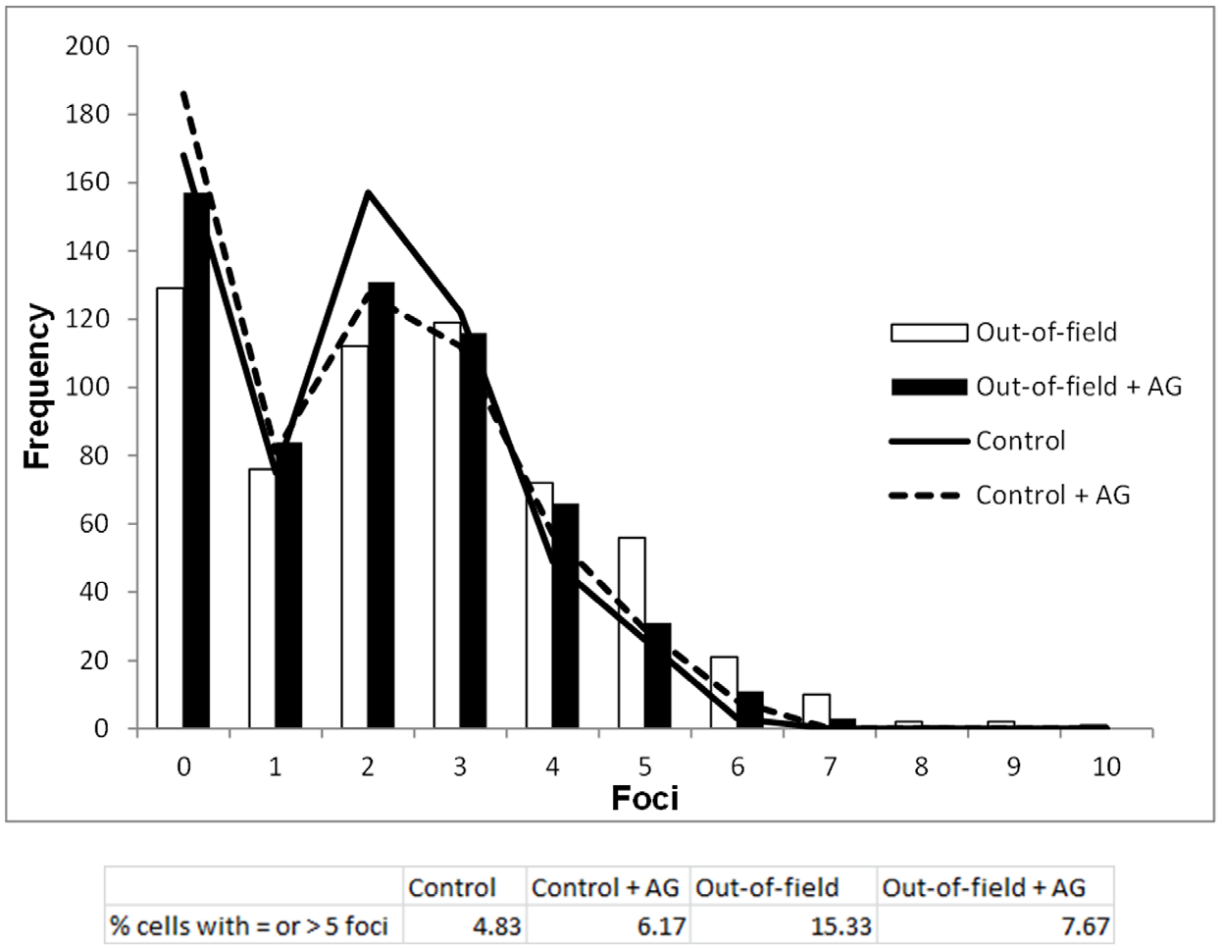
Frequency distribution of 53BP1 foci 24 hours following modulated radiation field exposure in the presence or absence of AG. Data are shown for AG0-1522B cells out-of-field with (Solid bars) and without (open bars) treatment with 20 µM AG. Distributions for non-irradiated controls treated with (dashed line) and without (solid line) AG are also included. The percentages of cells with greater than or equal to five foci are included in table format for clarity.

The role of communication in modulated exposures was further investigated by determining the DNA damage response via 53BP1 measurement with the addition of the inducible nitric oxide synthase (iNOS) inhibitor Aminoguanidine (AG). The Frequency distribution of foci for non-irradiated and out-of-field cell populations in the presence or absence of AG at 24 hours are shown in figure 10. Following 24 hours AG0-1522B cells without pre-treatment with AG showed a 10.5% increase in cells with 5 or more foci when compared to non-irradiated controls. In the presence of AG a notable reduction was observed in the percentage of cells with 5 or more foci with only 1.5% increase compared with the non-irradiated controls. By grouping observations together into a combined out-of-field population at 24 hours in the presence of AG (+AG), it was observed that the frequency distribution had a similar dispersion index when compared with non-irradiated control cells treated with AG (dispersions of 1.41±0.09 for non-irradiated cells in the presence of AG, and 1.37±0.09 for out-of-field cells in the presence of AG). Testing these distributions against non-irradiated controls cells in the absence of AG (−AG), using a two-tailed Z-score test, determined no significant difference between the populations suggesting complete abrogation of increased dispersion observed within out-of-field cells in the absence of AG treatment. It is noteworthy that while the addition of AG appears to give a slight increase in dispersion in control samples this difference was not significant.

## Discussion

With the development of advanced radiotherapy modalities such as IMRT it is of increasing importance to gain understanding in the effects of spatially modulated radiation exposures. The majority of radiation biology research has traditionally been performed in biological models that have been uniformly irradiated. In the current investigation we determined the DNA damage response via induction of 53BP1 and γH2AX foci following exposure to a simple modulated radiation field whereby 50% of the flask was shielded and investigated the spatial distribution of DNA damage across the modulated field. This work adds to previous studies that have outlined cellular responses to modulated fields [2]–[7] and in agreement with previous investigations suggests an important role for intercellular communication within the DNA damage response following modulated exposure.

In the present investigation we have shown a significant elevation in the average number of 53BP1 foci within the out-of-field region following exposure to a 1 Gy modulated field. For AG0-1522B and DU-145 cells a 1.6 and 1.5 fold increase in the average number of foci was observed out-of-field when compared with non-irradiated controls (fig. 3). This is consistent with an investigation by Hu *et al* [17] that observed a 2 fold increase in dsb in bystander areas following alpha particle irradiation. The response reported here was observed to be spatially dependent with a greater increase in the average number of foci 1 cm from the shielding edge. The increased foci number out-of-field was not limited to 53BP1 foci as analysis of γH2AX in the AG0-1522B cells showed comparable results (fig. 5). Throughout the investigation a rapid induction of DNA damage foci within regions out-of-field could be detected 30 minutes following modulated radiation field exposure. This is in agreement with previous investigations [16]–[17] that have highlighted rapid induction of DNA dsb in bystander areas using alpha-particle and medium transfer methods. Differential repair kinetics following the initial onset of DNA damage were observed in specific spatial regions out-of-field (fig. 8 C–D) with regions further from the shielding edge demonstrating a delayed response with constant elevation above non-irradiated control cells up to 6 hours after irradiation.

Following 24 hours after irradiation there is a suggestion of elevation in the average foci number compared with the non-irradiated controls across the entire out-of-field region. Further analysis of the frequency distribution of foci within regions out-of-field showed 10.5% increase, above non-irradiated controls, in number of cells containing 5 of more foci. Comparisons of dispersions indexes provide evidence that two populations of responding cells exist (fig. 6) containing a subset of cells with persistent elevated DNA damage compared with the non-irradiated controls. Taken together these results are consistent with work by Tartier *et al* [19] with a 12% increase, above the non-irradiated controls, in the number of bystander cells with greater than 4 foci 3 hours following irradiation. Within our current investigation the differences in the dispersion index were observed to be spatially dependent with the areas closer to the in-field region showing a greater difference in dispersion to the non-irradiated control cells. The increased levels of residual foci are also in line with the increased cell killing observed in the out-of-field area relative to the scattered dose [4]–[5]. Generally it is assumed that residual unrepaired dsb are responsible for lethality after acute radiation exposure [23]–[24]. For the bystander cells it also suggests that a continual increased production of DNA damage alongside repair (Figure 8), probably via accumulation of damage causing replication fork stalling in S-phase cells [20] leads to an increased probability of cell death.

Alongside the observed out-of-field response there is evidence of a diminished induction of foci in-field following modulated radiation field exposure when compared with uniformly irradiated cells. Exposure to a 1 Gy uniform radiation field rapidly increased the induction of 53BP1 and γH2AX foci 30 minutes following irradiation to greater than 20 foci per cell (fig. 2). Rapid induction of 53BP1 and γH2AX foci to the site of DNA damage following irradiation has been reported previously [21]–[22]. In response to a modulated radiation field a non-uniform response was observed in-field with a significant decrease in the average number of 53BP1 (fig. 3) and γH2AX (fig. 5) foci compared with a 1 Gy uniform exposure. Similarly to the out-of-field region the in-field DNA damage response is spatially dependent with a greater difference observed within areas closer to the centre of the slide. The decrease in the average number of foci in-field differs from what would be predicted when compared with the physical dose profile (fig. 1) and was independent on the percentage area irradiated (fig. 7). Following 24 hours after irradiation a differential dispersion index was observed between the in-field and uniform exposure suggesting two populations of responding cells exist within the in-field region within AG0-1522B cells. An increase in the average foci number compared with the uniform field may be due to increase in complexity of dsb within a population of in-field bystander responding cells that require a longer period of time to repair. However the DU-145 cells displayed no significant difference in the average number of foci between the uniform and in-field region 24 hours after irradiation suggesting that this response is specific to the normal fibroblast cell line.

Inhibition of cellular secreted intercellular communication was shown to abrogate the response both in-and out-of-field. Intercellular communication has been previously observed to have an important role in mediating the cellular response following modulated radiation fields [2], [4]–[5]. Investigations from our own laboratory have previously observed that inhibition of intercellular communication during exposure to modulated field was able to abrogate the decrease in cell survival out-of-field [4]–[5]. In the current investigation inhibition of cellular secreted communication between the in-and out-of-field regions not only abrogated the increase in DNA damage foci out-of-field but restored the in-field response to levels comparable with the uniform response.

In agreement with previous studies we have illustrated a role for reactive nitrogen species (RNS) within the out-of-field response to modulated radiation fields. The role of RNS within the bystander response has been reported previously [25]–[29]. Investigations from our laboratory have highlighted the importance of RNS in the out-of-field response with addition of AG, an (iNOS) inhibitor, resulting in increased cell survival in regions out-of-field [4]–[5]. The present study demonstrates a role for RNS in the residual levels of DNA damage out-of-field as inhibition of RNS production through AG treatment led to a reduction in the percentage of cells with elevated levels of 53BP1 foci 24 hours following modulated field exposure. Further statistical analysis illustrated that the increased dispersion observed in the out-of-field region when compared to non-irradiated controls was completely abrogated with the addition of AG suggesting inhibition of the bystander effect. This is in consistent with work by Shao *et al* [25] that reported a significant reduction of micronuclei formation within AG0-1522B bystander cells treated with AG.

The dependence upon intercellular communication within the DNA damage response indicates a possible role for radiation induced bystander signalling. The radiation-induced bystander effect describes the response of non-irradiated cells to signals produced by neighbouring cells that have been directly irradiated [8]. Increased DNA damage as a result of radiation induced bystander signalling has been reported previously [14]–[20]. There are two accepted mechanisms through which radiation-induced bystander effects are mediated: gap junction intercellular communication (GJIC) and through the secretion of factors by irradiated cells into the culture medium [8]. The experiments in the present study were performed using the non-confluent cells; therefore, only the radiation-induced bystander effect mediated through the release of soluble factors from irradiated cells could be investigated. The exact biological mechanism responsible for the radiation-induced bystander effect is complex and several molecular pathways have been shown to be involved [8].

In this study, for the first time, we report significant differences between the in-and out-of-field DNA damage responses of cells exposed to modulated radiation when compared with uniformly irradiated and non-irradiated control cells using a kV X-ray source. There was evidence for spatial dependence with a greater divergence from a uniform response observed in regions closer to the in-and out-of-field borders. The response observed was consistent for both cell lines investigated and with two markers of DNA damage. The current investigation provides further suggestion for an important role of the radiation-induced bystander effect in modulated exposures, where dose gradients are present, and may inform the refinement of conventional radiobiological models to assist the optimization of advanced radiotherapy treatment plans.
